# Probiotics in Asthma and Allergy Prevention

**DOI:** 10.3389/fped.2017.00165

**Published:** 2017-07-31

**Authors:** Maurizio Mennini, Lamia Dahdah, Maria Cristina Artesani, Alessandro Fiocchi, Alberto Martelli

**Affiliations:** ^1^Division of Allergy, Bambino Gesù Children’s Hospital, Vatican City, Vatican City; ^2^Department of Pediatrics, Salvini Hospital, Milan, Italy

**Keywords:** probiotics, asthma, allergy, prevention, children

## Abstract

Interest in probiotic research and its potential benefits in infant foods are relatively recent but significantly increasing. The evolution of the knowledge in the last 20 years demonstrated that alterations in the microbiome may be a consequence of events occurring during infancy or childhood, including prematurity, cesarean section, and nosocomial infections. Several pieces of evidence prove that a “healthy” intestinal microbiota facilitates the development of immune tolerance. Interventional studies suggest that probiotics could be protective against the development of many diseases. Nevertheless, many factors complicate the analysis of dysbiosis in subjects with food allergy. Comparison in-between studies are difficult, because of considerable heterogeneity in study design, sample size, age at fecal collection, methods of analysis of gut microbiome, and geographic location. Currently, there is no positive recommendation from scientific societies to use pre- or probiotics for treatment of food allergy or other allergic manifestations, while their use in prevention is being custom-cleared. However, the recommendation is still based on little evidence. Although there is valid scientific evidence *in vitro*, there is no sufficient information to suggest the use of specific probiotics in allergy and asthma prevention.

## Introduction

The 2001 FAO/WHO definition of probiotics (“live microorganisms that, when administered in adequate amounts, confer a health benefit on the host”) has been widely adopted by regulatory agencies, such as Codex alimentarius, the European Food Safety Authority (EFSA), scientists, industry, and consumers. Everyone agrees that a specific probiotic strain should have been investigated in properly controlled studies to confer a specific benefit before claiming the existence of such benefit. If this is not fulfilled, the only allowed claim would be “contains probiotics.” Studies using probiotics or prebiotics have been generally designed as exploratory and were not sufficiently designed to fulfill the criteria for substantiation of a health claim under the current regulation by EFSA (1).

In order to recommend specific probiotics or a mixture of probiotic strains for allergy prevention, they must prove to reduce the risk of later allergies when given to the pregnant or breast-feeding mother or directly to the infant.

Interest in probiotic research and its potential benefits in infant foods is quite recent, but significantly increasing. According to a recent bibliometric analysis, the total number of documents published on probiotics in pediatrics over the period 1994–2014 was 2817. Research production on probiotics in pediatrics showed a 90-fold increase during the study period. Approximately 22% of articles originated from USA and has the greatest share (2). The top 10 cited articles over the past two decades revealed that the majority of most important articles focused on the role of probiotics in the treatment of allergy and diarrhea in children. In Table 1, we summarize the main mechanisms of action of probiotics.

**Table 1:** Mechanisms of action of Probiotics.

Microbiological action	Epithelial action	Immunological action
– Modulation of the composition of the microbiota – Competitive adhesion to the receptors with the prevention of pathogens invasion – Production of bacteriocin with prevention of growth of pathogens	– Modulation of the epithelial cell barrier – Expression of the tight junction proteins – Short chain fatty acids with improvement of epithelial barriers and anti-inflammatory action	– Innate immunity modulation (maturation dendritic cells) – Modulation of Th1/Th2 rate – Increase of number and activity of T regulatory cells

## Probiotics in Pediatrics

In the last 20 years, it became clear that events occurring during infancy or childhood, including prematurity, cesarean section, and infections, influence the microbiome. Microbiome alterations have been associated with infantile colic, necrotizing enterocolitis, asthma, atopic diseases, diabetes, mood disorders, and autism spectrum disorders (3).

Interventional studies suggest that probiotics could prevent or reduce the severity of some of these diseases, but the biological mechanisms—and the optimal intervention for each—remain poorly understood.

## Probiotics for Allergy Prevention

A “healthy” intestinal microbiota facilitates the development of immune tolerance (4, 5). Earlier studies showed that gut-associated lymphoid tissues (GALT), including Peyer’s patches, are poorly developed or absent in germ-free mice (6, 7). It was shown that the introduction of *Bacteroides fragilis* into the lower gut of germ-free mice in the neonatal period could lead to a redevelopment of GALT and induction of tolerance (8). It was also proven that the inability to establish an effective immune tolerance early in life increases the host’s risk of developing allergic and inflammatory diseases (9). For example, mice raised in a sterile environment show reduced immunoglobulin A and interleukin (IL)-10 producing T regulatory (T_reg_) cells and are unable to develop oral antigenic tolerance (7, 10, 11). Segmented filamentous bacteria and Clostridium species, particularly clusters IV and XIVa, promote the development of IL-17-producing T cells and T_reg_ cells, respectively (12, 13). Furthermore, the gut microbiota of food allergic mice—but not of tolerant ones—transmitted susceptibility to food allergy when transferred into germ-free mice (14).

Many factors complicate the analysis of dysbiosis in subjects with food allergy. Comparisons between studies are difficult, because of heterogeneity in study design, sample size, age at fecal collection, methods of analysis of gut microbiome, and geographic location (15). Nevertheless, evidence of gut dysbiosis in food allergy is evolving with time, aided by increasing availability of new techniques. Studies relying on bacterial cultures showed that infants allergic to cow’s milk had higher total bacteria and anaerobic counts (16), but this finding was not consistent across studies (17) and no association could be established between culturable gut bacteria and sensitization to food, including milk, casein, egg, peanut, and hazelnut (18).

The hypothesized mechanisms by which the commensal microbiota influences the outcome of the allergic response are manifold (19). Intestinal bacteria can modulate the innate lymphoid cells, directly acting on T_regs_ through their toll-like receptors (TLRs). Commensal microbiota promotes the differentiation of induced T_regs_ (iT_reg_) from naïve CD4^+^ T-cells by a T_reg_ intrinsic, TLR- and myeloid differentiation primary response gene 88 (MyD88)-dependent mechanism (20, 21).

Another mechanism by which the commensal flora promotes tolerance is the production of short chain fatty acids (SCFAs), generated by bacterial fermentation of dietary fibers. SCFA act on T cells *via* a G-protein-coupled receptor (GPR43) and protect mice from intestinal inflammation by expanding colonic T_reg_ cells (22). SCFAs also promote the generation of intestinal T_reg_ cells from naïve CD4^+^ T cells by T-cell intrinsic epigenetic mechanisms (23). Butyrate, a SCFA known as histone deacetylase inhibitor, increases Foxp3 protein acetylation conferring increased stability and enhanced suppressive function on *de novo* generated intestinal iT_reg_ cells (24). A high fiber diet protects against allergic airway inflammation by altering the composition of the flora, leading to increased Bacteroidetes and decreased Firmicutes, and resulting in increased circulating levels of SCFAs (25).

## Probiotics for Prevention of Asthma and Eczema

In general, preventive strategies for asthma and allergic disorders have been proposed in 2014 (26):

(1) General health education: avoidance of tobacco smoke exposure during pregnancy and after birth.
(2) Primary prevention for infants at higher risk. Several longitudinal birth cohort studies have clearly demonstrated an increased risk of allergic manifestations if one or two parents are or have been affected themselves.
(3) Secondary prevention strategies for children who have already developed allergic sensitization or the first manifestations of allergic diseases; those strategies aim to reduce the incidence of clinical manifestations, such as rhinitis, food allergy, or asthma.

Pre-clinical studies have shown that modifying the microbiota could modulate the global immune response of the host, thus reducing sensitization and allergic inflammation (7, 11). Many studies have suggested the hypothesis that pre- and probiotics might be protective for asthma.

The inhalation of allergens stimulates the innate immune system to release cytokines which promote antigen expressions on CD4^+^ T-cells and activate the antigen-presenting cells and the T cells to produce Th2 responses (27, 28). Th2 cytokines (IL-4, IL-5, IL-9, and IL-13) induce asthma-like changes in the airways and lung parenchyma, as airway eosinophilia, pulmonary lymphocytosis, mastocytosis, alternative macrophage activation, and epithelial cell proliferation with goblet cell hyperplasia. Previous studies have shown that matrix metalloproteinases, members of a family of enzymes that cleave extracellular matrix proteins, are implicated in many inflammatory conditions (29). Specifically, in asthma, MMP9 levels are significantly increased (30). Treatment with LGG has been shown to decrease MMP9 expression in lung tissue and to inhibit inflammatory cell infiltration. In addition, in OVA-sensitized mice, LGG reduced OVA-specific IgE levels in serum, suppressed the airway hyper-responsiveness to methacholine and decreased the number of infiltrating inflammatory cells and Th2 cytokines in bronchoalveolar lavage fluid and serum (31). Similar results have been reported with other probiotics (32).

Specifically, in pediatric asthma, LGG was reported to reduce the concentration of exhaled nitric oxide among 4- to 7-year-olds (33), but these results could not be replicated (34).

Early administration of *Lactobacillus reuteri* to infants did not result in a reduction of asthma [RR 1.16 (0.33–4.10)], nor did *Lactobacillus rhamnosus* HN001 [RR 0.95 (0.62–145)] or *Lactobacillus paracasei* spp. *paracasei F19* [RR 1.05 (0.39-2.81)] (35–37).

Better results have been obtained with probiotic bacteria based on *in vitro* modulation of cytokine production. *Bifidobacterium bifidum, B. lactis*, and *Lc. lactis* were shown to have a good IL-10-inducing capacity and to exert a significant inhibition of Th2-related cytokines IL-5 and IL-13 (38–40). Administered perinatally in a selected combination, they reduced the development of eczema up to the age of 2 years. Their beneficial effect does not reach the age of 6 years and does not lead to primary prevention of asthma.

A systematic review of randomized trials assessing the effects of any probiotic administered to pregnant women, breast-feeding mothers, or infants demonstrated that probiotics could reduce the risk of eczema in infants (41). The certainty in the evidence is low or very low because of the risk of bias, inconsistency and imprecision of results, and indirectness of available research.

As underlined in two recent reviews, replication of the promising results in collaborative well-coordinated multicentre harmonized studies with multidisciplinary expertise in pediatrics, immunology, and microbiology would, thus, be of great importance to enable future evidence-based implementation (42).

A more prolonged gut microbiota management could achieve a long-lasting impact (43, 44).

## Guidelines Recommendations: Over to Scientific Societies

– The European Academy of Allergy and Clinical Immunology (EAACI) stated in its food allergy and anaphylaxis guidelines on primary prevention of allergy, that “there is no evidence to recommend prebiotics or probiotics or other dietary supplements based on particular nutrients to prevent food allergy” in at risk groups and in the general population (grade of recommendation B) (45).
– The Nutrition Committee of the European Society of Paediatric Gastroenterology, Hepatology and Nutrition (ESPGHAN) concluded 2011 after a systematic literature review on the effect of infant formula supplemented with prebiotics or probiotics on the preventive effect on allergy, that “there is too much uncertainty to draw reliable conclusions from the available data” (46).
– The World Allergy Organization (WAO) suggested 2015 on their guidelines on the prevention of allergy to consider using probiotics in:
  (a) women pregnant with children with high risk for allergy,
  (b) women who breastfeed infants at high risk of developing allergy, and
  (c) infants at risk of developing allergies, because there is a net benefit resulting in primary prevention of eczema (47).

## Conclusion

There is no positive recommendation from any scientific community to use specific probiotics for the prevention of food allergy or other allergic manifestations (48), but their use in prevention as a whole class has widespread in clinical practice (49, 50). We are more open to the use of probiotics than in the past, but the recommendation is based on little evidence. Although there is valid scientific evidence *in vitro*, there is no sufficient information to suggest that the use of probiotics is effective in preventing allergy and asthma. At this point, it seems necessary to understand more precisely the microbiota composition of healthy humans. Only by identifying the specific changes, we would realize that the “ideal probiotic,” able to prevent or fight specific dysbiosis of specific disease. Future studies will take stock of state-of-the-art methods for the evaluation of the microflora to better define the indications, the probiotic strains, and the type of prebiotic used.

## Author Contributions

MM, AF, and AM drafted the manuscript and provided critical input to the manuscript, and all authors approved the final version. LD and MA revised and approved the manuscript in this version.

## Conflict of Interest Statement

The authors declare that the research was performed in the absence of any commercial or financial relationships that could be construed as a potential conflict of interest. The reviewer, CM, and handling editor declared their shared affiliation, and the handling editor states that the process nevertheless met the standards of a fair and objective review.
